# Size-Controlled Fabrication of Alginate Hydrogel Microbeads Optimized for Lipase Entrapment

**DOI:** 10.3390/gels11090710

**Published:** 2025-09-04

**Authors:** Dong Han Kim, Jeong Eun Cha, Dojin Kim, Sang Hyun Lee

**Affiliations:** Advanced Materials Program, Department of Biological Engineering, Konkuk University, Seoul 05029, Republic of Korea; edike1211@naver.com (D.H.K.); cje0430@konkuk.ac.kr (J.E.C.); dojin135@naver.com (D.K.)

**Keywords:** size-controlled microbead, alginate hydrogel, lipase, entrapment

## Abstract

Enzyme entrapment in alginate hydrogel microbeads is an effective method of immobilization for industrial applications, but many fabrication methods for alginate microbeads involve oil, organic solvents, or high temperatures that reduce enzymatic activity. In this study, we employed an oil- and solvent-free gas-shearing technique to prepare alginate microbeads for the entrapment of *Candida rugosa* lipase (CRL), thereby minimizing thermal- and solvent-induced inactivation. To enhance immobilization efficiency and reusability, the effects of gas flow rate, alginate concentration, and cross-linking metal ions were systematically investigated. CRL entrapped in Ba- and Fe-alginate microbeads showed superior immobilization yield, activity retention, and activity recovery compared with CRL entrapped in conventional Ca-alginate microbeads. Notably, both Ba- and Fe-alginate microbeads exhibited significantly enhanced stability, with half-lives up to 127-fold greater than that of free CRL at 60 °C, and maintained substantially higher pH stability across the tested range. Ba-alginate microbeads provided greater pH stability and substrate affinity, whereas Fe-alginate microbeads demonstrated enhanced thermal stability and catalytic turnover. These findings highlight gas-shearing as a scalable and gentle fabrication method for producing high-performance alginate microbeads with tunable properties, making them suitable for enzyme entrapment in diverse biocatalytic applications.

## 1. Introduction

Enzymes, predominantly proteins, act as biocatalysts that accelerate a wide range of biochemical reactions, including degradation, oxidation–reduction, and molecular transformations. Owing to their catalytic properties, they are widely used in industrial sectors, such as food processing, pharmaceuticals, biotechnology, and environmental engineering. Among industrial enzymes, lipases account for approximately 10% of global enzyme production [1] and catalyze the hydrolysis and synthesis of esters with high specificity, supporting applications in food manufacturing, chemical synthesis, cosmetics, detergents, biodegradable plastics, biofuel production, and waste oil treatment [2]. In addition to these established applications, lipases are being increasingly explored for biomedical applications, including drug delivery systems and biosensors, because they function under mild conditions and exhibit chemo-, regio-, and enantioselectivity, which are advantageous for therapeutic and diagnostic development [3]. However, the practical use of lipases in industrial and biomedical processes often requires prolonged operation under elevated temperatures, varying pH conditions, and repeated reaction cycles. These operational demands highlight the need for robust immobilization systems capable of preserving enzyme activity and enabling efficient recovery and reuse.

Enzyme immobilization is a widely adopted strategy for improving operational stability, facilitating recovery, and enabling the repeated use of biocatalysts in industrial and biomedical processes [4,5]. Immobilization can be achieved through various methods, including adsorption, covalent binding, and entrapment, each of which has distinct advantages and limitations [6]. Among these, entrapment in alginate hydrogels is particularly attractive because of their biocompatibility, low toxicity, mild gelation conditions, and simple processing, which make them especially suitable for biomedical applications. Owing to these advantages, alginate hydrogels have been employed not only for lipases but also for various other enzymes such as peroxidases, oxidases, proteases, and catalases, demonstrating their broad applicability as versatile immobilization carriers [7,8,9]. Alginate hydrogels are typically prepared via ionic cross-linking with multivalent cations such as Ca^2+^, producing hydrogel beads capable of physically entrapping enzymes while maintaining their native conformation [10]. However, conventional Ca-alginate beads often suffer from limited mechanical stability and low reusability under processing conditions, particularly when exposed to hydrophobic substrates or mechanical agitation [11]. Bead size critically affects immobilization performance. Millimeter-scale beads can load more enzymes and provide higher durability, suiting them for industrial reactors, whereas microbeads offer a larger surface-to-volume ratio and better mass transfer, making them advantageous for biomedical use. Fabricating beads across different size ranges broadens application potential, but the weak structural strength of microbeads limits their reuse and long-term stability [12,13,14]. Therefore, the development of alginate microbeads with high mechanical strength is the most desirable goal and can enhance the applicability of alginate hydrogels as support for enzyme entrapment.

Several techniques have been developed to produce alginate hydrogel microbeads for enzyme entrapment, including emulsion, electrospraying, and spray drying. However, these methods present fundamental limitations because the gelation conditions required for fabricating alginate microbeads are often incompatible with enzyme entrapment, frequently leading to enzyme inactivation. The emulsion method, which disperses an aqueous alginate solution into an oil phase, followed by cross-linking, can generate microbeads of various sizes; however, it often leaves residual surfactants or oils, requires extensive washing, and provides limited size control [15]. Electrospraying enables precise microbead size control in the nano- to microscale range using high-voltage electric fields [16,17]; yet, it typically involves organic solvents that may inactivate enzymes, offers relatively low throughput, and demands complex high-voltage equipment [18]. Spray drying can rapidly produce microbeads by atomizing polymer solutions into heated air streams but is generally unsuitable for heat-sensitive enzymes because of the high temperatures involved [19,20]. In contrast, recently developed gas-shearing is a solvent- and oil-free technique that uses an inert gas (e.g., nitrogen) to apply a shear force to droplets at the nozzle tip, lowering the surface tension and promoting rapid detachment to form uniform microbeads [21]. This method enables continuous production with high throughput, precise control of the microbead size by adjusting the gas flow rate and solution parameters, and minimal post-processing, thereby avoiding conditions that could reduce enzyme activity [22]. In addition, its oil-free nature reduces downstream washing requirements, lowering overall processing costs, and the technique can be scaled by operating multiple units in parallel or integrating into continuous flow systems, highlighting its industrial potential.

Although gas-shearing has been explored in previous studies, most applications have focused on cell entrapment or drug delivery systems, with limited attention to optimizing conditions for enzyme entrapment [21,23]. To our knowledge, no systematic study has demonstrated how gas-shearing can be tailored to simultaneously enhance catalytic activity and mechanical robustness of enzyme-entrapped alginate microbeads, which remains a critical barrier to their practical use. Addressing this gap is particularly relevant for industrial biocatalysis, where scalable and economical immobilization methods are needed to enable practical applications.

When alginate microbeads are produced via gas-shearing without additional structural reinforcement, their small size, combined with the intrinsic mechanical weakness of alginate, can lead to poor durability and reusability. These limitations can be overcome by optimizing the fabrication parameters such as the gas flow rate, alginate concentration, and type of cross-linking metal ions. The choice of the cross-linking ion is particularly important because it determines the strength and architecture of the gel network. Although Ca^2+^ is the most commonly used ion for alginate gelation, alternative cations such as Ba^2+^ and Fe^3+^ can form stronger and more stable networks, potentially improving microbead robustness [24]. Although such optimization can yield alginate microbeads with high robustness, studies applying this approach to enzyme entrapment remain limited. The present study aims to address this limitation.

In this study, we developed a custom-designed gas-shearing apparatus to produce size-controlled alginate microbeads and evaluated its suitability for enzyme entrapment. *Candida rugosa* lipase (CRL) was selected as a model enzyme because of its broad industrial and biomedical applications. The effects of gas flow rate, alginate concentration, and cross-linking metal ion type on microbead morphology, immobilization yield, catalytic activity, thermal stability, pH stability, and reusability were investigated. This investigation aims to establish gas-shearing as a scalable and gentle fabrication method for producing alginate microbeads with tunable properties, providing a versatile platform for enzyme entrapment in diverse biocatalytic applications.

## 2. Results and Discussion

### 2.1. Entrapment of CRL into Size-Controlled Alginate Hydrogel Microbeads

#### 2.1.1. Development of a Gas-Shearing Apparatus for Preparing Alginate Hydrogel Microbeads

**Figure 1:**
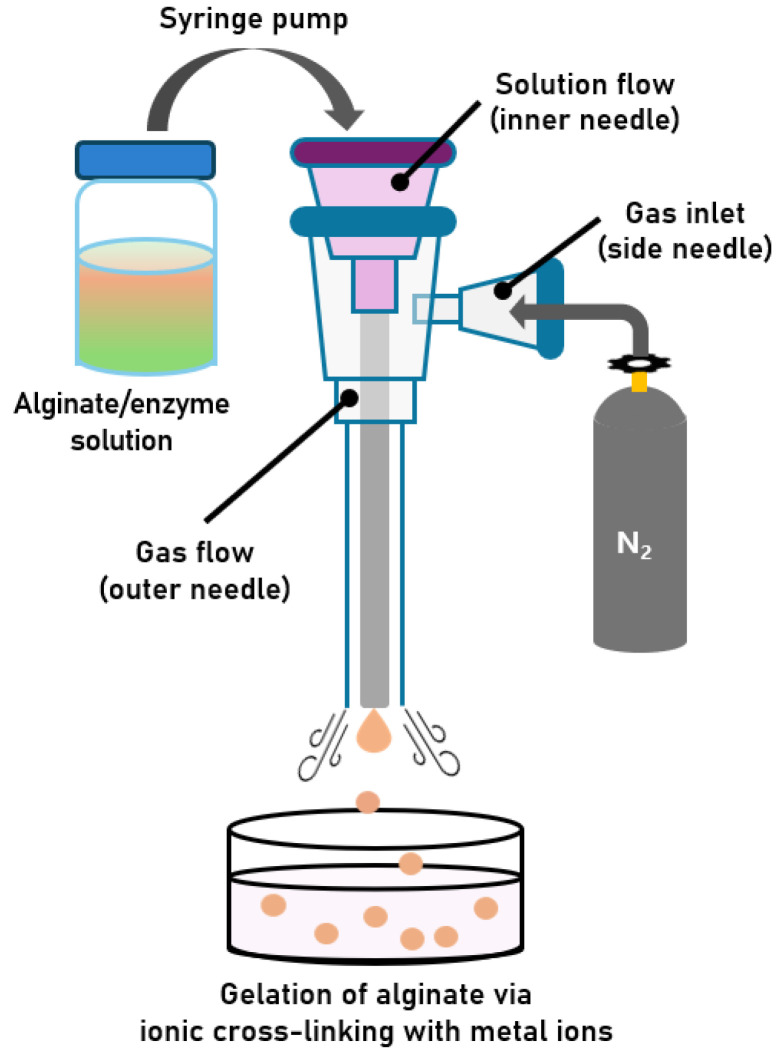
Schematic illustration of the gas-shearing process for fabricating size-controlled alginate microbeads containing an enzyme.

As shown in Figure 1, a custom-designed gas-shearing apparatus was constructed with modifications based on previously reported designs [25,26]. The apparatus consists of three needles. The inner needle serves as the channel through which the alginate solution is delivered from a syringe into a bath solution containing cross-linking metal ions (e.g., Ca^2+^). This inner needle is concentrically enclosed by an outer needle, which functions as a pathway for nitrogen gas flow. An additional side needle, positioned adjacent to the outer needle, serves as the gas inlet, through which nitrogen gas enters and flows downward through the outer needle. The nitrogen gas emitted from the outer needle exerts a shear force on the droplet emerging from the tip of the inner needle. This shearing action reduces the surface tension of the droplet, facilitating rapid detachment of the droplet from the needle tip. Increasing the gas flow rate enhances the shearing force, leading to a further reduction in surface tension and the formation of smaller alginate microbeads. Here, surface tension resists droplet deformation, while gas shear promotes detachment; when shear overcomes surface tension, smaller droplets are generated [21]. Therefore, by controlling the nitrogen gas flow rate, the apparatus enables the production of size-controlled alginate microbeads. Encapsulation of enzymes within alginate microbeads can be readily achieved using an alginate/enzyme mixture instead of an alginate solution. In this study, CRL was selected as the model enzyme and entrapped within alginate microbeads using the developed gas-shearing system.

#### 2.1.2. Effects of Gas Flow Rate on the Microbead Size and the Activity of Entrapped CRL

When droplets of the alginate/CRL solution, released at a controlled rate, come into contact with the CaCl_2_ solution, gelation is initiated through an ionic exchange reaction between Na^+^ ions from the sodium alginate and Ca^2+^ ions. The carboxylate groups (–COO^−^) in the guluronic acid residues (G blocks) of alginate interact with Ca^2+^ to form a two-dimensional cross-linked structure, commonly described by the egg-box model [27]. During this process, the CRL co-dissolved in the alginate solution becomes entrapped within the size-controlled alginate microbeads. As shown in Table 1, increasing the gas flow rate from 0.5 to 2.0 L/min resulted in a substantial reduction in the mean diameter of CRL-entrapped alginate microbeads, from 893 µm to 247 µm. The corresponding particle size distribution profiles obtained using a particle size analyzer are presented in Figure S1. In parallel with size reduction, the total number of microbeads formed increased markedly from 122 to 14,500. These results indicated that the gas-shearing apparatus developed for size-controlled alginate microbead fabrication was equally effective in producing CRL-entrapped alginate microbeads, confirming successful entrapment of CRL within the size-controlled carriers.

The size of the alginate microbeads can significantly influence the immobilization efficiency of entrapped CRL, including key parameters such as immobilization yield, activity retention, and activity recovery [28]. The immobilization yield reflects the proportion of the initially added CRL that was successfully entrapped within the alginate microbeads. This is calculated by dividing the amount of immobilized enzyme by the initial amount of free enzyme added to the alginate solution and multiplying the result by 100 to obtain a percentage [29,30]. As the microbead diameter decreases, the internal volume available for enzyme entrapment decreases, thereby limiting the total amount of enzyme that can be incorporated [12]. As shown in Table 1, the immobilization yield decreased from 44.9% to 20.3% as the average microbead diameter was reduced from 893 µm to 247 µm. This trend is well described by the following linear regression model:

(Immobilization yield) = 0.0358 × (mean diameter of microbead) + 14.0(1)

The high correlation coefficient (R^2^ = 0.964, *n* = 5) suggested a strong dependence of the immobilization yield on microbead size. These findings indicated that although the gas-shearing apparatus enabled precise control over microbead size, smaller beads inherently limited the enzyme loading capacity owing to reduced internal volume.

Activity retention refers to the percentage of enzymatic activity retained after immobilization, relative to the activity of the free enzyme. It is defined as the ratio of the specific activity (SA) of the immobilized enzyme (U/mg) to that of the free enzyme (U/mg) multiplied by 100 [29,30]. As shown in Table 1, the activity retention increased with decreasing microbead size. This trend can be attributed to the improved mass-transfer characteristics of the smaller microbeads. Specifically, smaller microbeads possess a higher surface-to-volume ratio, which promotes more efficient diffusion of the substrate into the microbead interior and rapid removal of the reaction products [13,14,29]. This reduces local product accumulation, which could otherwise inhibit enzymatic activity. In contrast, larger microbeads tend to entrap enzyme molecules deeper in the core, where limited substrate access or diffusion barriers may reduce the activity or lead to partial deactivation [31]. In smaller microbeads, the reduced diffusion path ensures more uniform substrate access and better exposure of the entrapped enzyme to the reaction medium, thereby enhancing the observed activity.

Activity recovery represents the overall effectiveness of the immobilization process in preserving enzymatic function. It is defined as the ratio of the total activity of the immobilized enzyme (U) to the total activity of the initially added free enzyme (U), multiplied by 100 [29,30]. This value can also be interpreted as the product of immobilization yield and activity retention, thereby reflecting both the amount of immobilized enzyme and the extent to which its catalytic function is retained. Therefore, activity recovery serves as a comprehensive indicator of immobilization efficiency and is particularly useful for evaluating the practical and economic feasibility of immobilization strategies. As shown in Table 1, the activity recovery ranged from 2.6% to 6.0% across different gas flow rates. Although this trend was not strictly monotonic, the highest activity recovery was observed with the smallest microbead size. This suggests that smaller microbeads, despite entrapping a smaller quantity of enzyme due to their limited internal volume, can provide a more favorable microenvironment for catalytic activity. The enhanced surface-to-volume ratio and shortened diffusion distances in smaller microbeads likely improved substrate accessibility and product removal, thereby contributing to the increased overall catalytic efficiency.

The reusability of the CRL-entrapped alginate microbeads was evaluated as an indirect measure of their mechanical stability. Microbeads prepared at the lowest gas flow rate (0.5 L/min), with the largest average diameter of 893 µm, could be reused up to two cycles. However, during washing prior to the third cycle, the microbeads dissolved in the washing buffer, indicating structural fragility under repeated handling. In the case of microbeads fabricated at a gas flow rate of 0.8 L/min (average diameter: 576 µm), only one reuse cycle was possible. The enzymatic activity increased after the first reuse, likely because of partial leakage of the entrapped lipase into the reaction medium. Microbeads produced at gas flow rates of 1.0 L/min or higher could not be reused, as they disintegrated during the initial washing step, suggesting insufficient mechanical strength at smaller sizes. Smaller microbeads are more susceptible to physical damage during vacuum filtration and washing because of their lower mechanical strength and higher surface sensitivity [32]. Additionally, the influence of the substrate should not be overlooked. The use of a hydrophobic substrate such as p-nitrophenyl butyrate (pNB) may further weaken the gel stability of Ca-alginate. Alginate is a hydrophilic hydrogel that is optimized for use in aqueous environments. The penetration of hydrophobic molecules into the gel may disrupt hydrogen bonds and electrostatic interactions within the polymer network [33]. In particular, the adsorption of pNB onto the microbead surface or within the internal gel matrix may lead to localized contraction, hardening, or microcracking [34]. Therefore, repeated substrate exposure could accelerate the loss of elasticity and structural integrity, with smaller microbeads being especially susceptible owing to their higher surface area and faster diffusion kinetics.

Although smaller alginate microbeads are generally advantageous for improving the enzymatic activity owing to their higher surface-to-volume ratio, they often exhibit poor reusability owing to their insufficient mechanical stability. To develop entrapment systems that achieve high catalytic efficiency and structural robustness, further optimization of the microbead fabrication conditions was pursued. In this context, a gas flow rate of 1.5 L/min was selected instead of 2.0 L/min to improve the process controllability and ensure consistent microbead morphology. Based on these conditions, subsequent experiments investigated the effects of two additional variables, namely, the concentration of alginate and the type of cross-linking metal ion, both of which are known to influence the structural properties of microbeads and the performance of the entrapped enzyme system.

#### 2.1.3. Effects of Alginate Concentration on the Activity and Reusability of Entrapped CRL

The concentration of alginate plays an important role in determining the structural and functional characteristics of entrapped enzymes, particularly their catalytic efficiency and reusability [28]. As shown in Table 2, increasing the alginate concentration from 3% to 5% decreased the activity recovery from 5.3% to 3.4%. This reduction is attributed to the formation of a denser gel matrix at higher concentrations, which may hinder mass transfer between the substrate and the entrapped CRL. Increasing the alginate concentration also resulted in a gradual increase in microbead size, from 282 μm at 3% to 399 μm at 5%. Microbeads prepared with 3% and 4% alginate disintegrated during the washing step after the initial reaction, rendering their reuse unfeasible. In contrast, the microbeads formed with 5% alginate maintained their structural integrity and enabled at least one additional reaction cycle. A residual activity of approximately 300% was observed during the second cycle, which may be attributed to partial microbead degradation and the consequent release of the entrapped CRL into the reaction medium. These findings suggest that although higher alginate concentrations enhance the mechanical stability of the microbeads, they may also limit catalytic performance by restricting substrate diffusion [35]. Considering this trade-off, 5% alginate was selected as the optimal condition because it offered a practical balance between mechanical robustness and catalytic performance. At this concentration, the microbeads maintained structural integrity during washing and reuse, while still retaining enzymatic activity.

#### 2.1.4. Effects of Cross-Linking Metal Ions on the Activity and Reusability of Entrapped CRL

Sodium alginate forms hydrogel networks through ionic interactions with divalent cations, a process typically described by the egg-box model. In this arrangement, each divalent ion bridges carboxyl groups from adjacent guluronic acid blocks to form planar junction zones, and the strength of these junctions depends on the relative binding affinity of the ions. Divalent cations such as Mg^2+^, Ca^2+^, Sr^2+^, and Ba^2+^—all belonging to Group 2 alkaline earth metals—interact with the G-block residues in alginate to form two-dimensional cross-linked structures [27]. These cations exhibit different binding affinities to alginate (Ba^2+^ > Sr^2+^ > Ca^2+^ > Mg^2+^), which strongly influence the structural and functional properties of the resulting microbeads. Stronger ionic interactions lead to tighter cross-linking, resulting in smaller microbead diameters, higher enzyme immobilization yields, and greater retention of enzyme activity [36,37,38]. This trend is partially attributed to the increasing ionic radii of the cations, which enhance their coordination with the carboxyl groups in alginate. Among the Group 2 divalent ions, Ba^2+^, having the largest ionic radius, is particularly effective in forming stable egg-box structures, followed by Sr^2+^ and Ca^2+^. In contrast, Mg^2+^, with its smaller size and limited bridging capacity, forms only weak interactions with alginate, leading to poor gelation or mechanically unstable microbeads [39,40].

**Figure 2:**
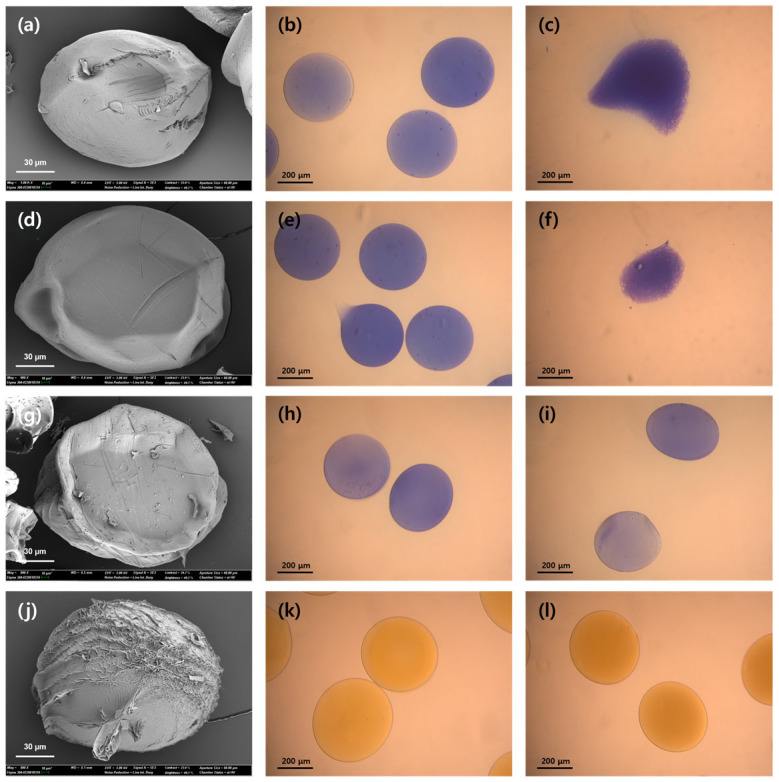
Scanning electron microscopy (left column) and optical microscopy images (middle: initial state, right: after reuse) of CRL-entrapped alginate microbeads cross-linked with different metal ions. (**a**–**c**): Ca-alginate microbeads (initial and after 1st reuse), (**d**–**f**): Sr-alginate microbeads (initial and after 4th reuse), (**g**–**i**): Ba-alginate microbeads (initial and after 4th reuse), (**j**–**l**): Fe-alginate microbeads (after 4th reuse). Ca-, Sr-, and Ba-alginate microbeads were stained with methylene blue to visualize their morphology.

As shown in Table 3, the average microbead diameter followed the order Ca^2+^ > Sr^2+^ > Ba^2+^, consistent with the trend in binding affinity [36,37]. Mg^2+^ ions failed to form microbeads owing to their insufficient cross-linking strength. Stronger cross-linking leads to more compact gel networks with reduced porosity, enhancing enzyme entrapment. Accordingly, the immobilization yield increased progressively from Ca-alginate to Ba-alginate microbeads, accompanied by improvements in both activity retention and activity recovery. These enhancements reflect improved mass transfer and stabilization of the enzyme within the tighter gel matrix. The enhanced cross-linking strength also contributed to improved reusability. The Ca-alginate microbeads (Figure 2c) disintegrated after a single reuse cycle, indicating poor mechanical stability. In contrast, the Sr-alginate microbeads (Figure 2f) maintained their structural integrity for up to four cycles, although they eventually collapsed. The Ba-alginate microbeads (Figure 2i) remained intact after four reuse cycles, demonstrating superior mechanical robustness. The swelling ratio followed the order Ca^2+^ > Sr^2+^ > Ba^2+^ (Table 3), reflecting increasing gel compactness and reduced internal water permeability [41].

Trivalent cations such as Fe^3+^ form distinct three-dimensional gel networks by simultaneously coordinating with three carboxyl groups on different alginate chains [42]. This multivalent coordination produces highly interconnected structures with greater cross-link density than the planar junctions formed by divalent ions, thereby generating denser and mechanically stronger gels. In addition, the high charge density and strong complexation ability of Fe^3+^ further reinforce the network, contributing to its robustness [41].

Among all the tested metal ion cross-linkers, Fe-alginate microbeads achieved the highest immobilization yield with superior activity retention and activity recovery (Table 3). These results are ascribed to the dense and robust gel matrix formed through trivalent coordination of Fe^3+^ with alginate chains. In contrast to the deformation observed in Ca- (Figure 2c) and Sr-alginate microbeads (Figure 2f), Fe-alginate microbeads maintained their structural integrity after four reuse cycles (Figure 2l), indicating mechanical stability comparable to that of Ba-alginate microbeads [24]. Interestingly, the Fe-alginate microbeads exhibited the highest swelling ratio among all samples, which may seem unexpected given their strong cross-linked networks [41]. This behavior is likely attributable to the rapid diffusion of Fe^3+^ ions during gelation, which results in an inhomogeneous internal structure. Specifically, a densely cross-linked outer shell may form, whereas the inner core remains relatively loosely cross-linked, allowing for greater water absorption upon rehydration [43]. Furthermore, it is possible that more extensive cross-linking led to greater dehydration during the drying process, which partially explains the high swelling ratio measured after drying. Similar findings have been reported, where Fe^3+^-alginate microbeads showed greater swelling and initial microbead size compared to Fe^2+^-alginate microbeads, even though their final dried size was smaller due to stronger dehydration [44].

After four reuse cycles, the residual activity of the lipase-entrapped microbeads was 77.7%, 63%, and 50% for Sr-alginate, Ba-alginate, and Fe-alginate, respectively. Although the Sr-alginate microbeads exhibited the highest residual activity, the structural deformation observed in Figure 2f suggests that enzyme leakage during repeated use may have caused overestimation of the retained activity. In contrast, both the Ba- and Fe-alginate microbeads maintained stable gel structures after four cycles (Figure 2i,l) and preserved more than 50% of their initial activity, confirming their suitability for repeated biocatalytic applications.

Overall, the results showed that the performance of CRL entrapped in alginate microbeads depends on both physical parameters, such as microbead size and alginate concentration, and chemical factors, particularly cross-linking metal ions. Smaller microbeads improved mass transfer and activity retention but often lacked mechanical stability, whereas higher alginate concentrations enhanced microbead integrity but reduced catalytic efficiency. Among the ions tested, Ba^2+^ produced the most compact and robust divalent gels, and Fe^3+^ formed dense three-dimensional networks with the highest immobilization yield and activity recovery. Both the Ba- and Fe-alginate microbeads maintained their structural integrity over multiple reuse cycles, highlighting the importance of metal ion selection along with optimized microbead size and polymer concentration to balance enzymatic activity, stability, and reusability.

### 2.2. Characteristics of CRL Entrapped in Alginate Hydrogel Microbeads

#### 2.2.1. Thermal Stability of Entrapped CRL

To evaluate the performance of immobilized enzymes, both operational stability (reusability) and thermal stability must be considered. CRL exhibits an optimal activity at approximately 37 °C [45] but undergoes rapid denaturation and loss of activity at higher temperatures. Because chemical reaction rates generally increase with temperature, enhancing the thermal stability of CRL would broaden its potential application range. Immobilization can improve enzyme thermal stability by increasing enzyme–matrix interactions and restricting enzyme flexibility within the confined space of the gel network. In this study, the thermal stability of CRL entrapped in Ba- and Fe-alginate microbeads, both of which exhibited high gel strength and sustained multiple reuse cycles, was compared with that of free CRL.

**Figure 3:**
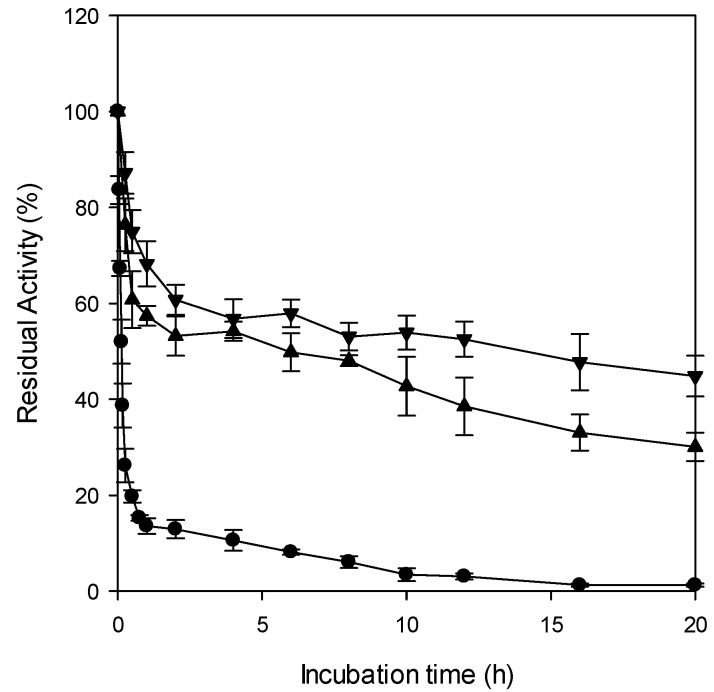
Thermal stability at 60 °C of free CRL (●) and CRL entrapped in Ba-alginate microbeads (▲) and CRL entrapped in Fe-alginate microbeads (▼).

The residual activity of free CRL and entrapped CRL was measured after incubation at 60 °C for up to 20 h (Figure 3). Free lipase lost more than 50% of its initial activity within the first 10 min, whereas CRL entrapped in Fe-alginate microbeads retained 52% of its initial activity even after 12 h. Based on the first-order enzyme deactivation kinetics, the half-lives of free CRL, CRL entrapped in Ba-alginate, and CRL entrapped in Fe-alginate were calculated to be 9 min, 13 h, and 19 h, respectively. These results indicate that free CRL exhibits very poor thermal stability at 60 °C, whereas entrapment markedly improves thermal resistance. Notably, the half-life of CRL entrapped in Fe-alginate microbeads was approximately 127-fold higher than that of the free CRL and around 1.5-fold higher than that of CRL entrapped in Ba-alginate. This superior thermal stability likely resulted from the stronger and more extensive cross-linking of Fe^3+^ ions with alginate, whose trivalent coordination forms a dense and thermally resilient gel network that helps maintain microbead integrity under heat and minimizes enzyme leaching from the matrix [43].

#### 2.2.2. pH Profile and pH Stability of Entrapped CRL

Understanding the pH dependence of enzyme activity and stability is essential for optimizing biocatalytic performance, as both parameters directly affect reaction rates and enzyme lifetimes under process conditions. Immobilization can alter the microenvironment surrounding the enzyme, potentially shifting its optimum pH and improving its stability over a broad pH range.

**Figure 4:**
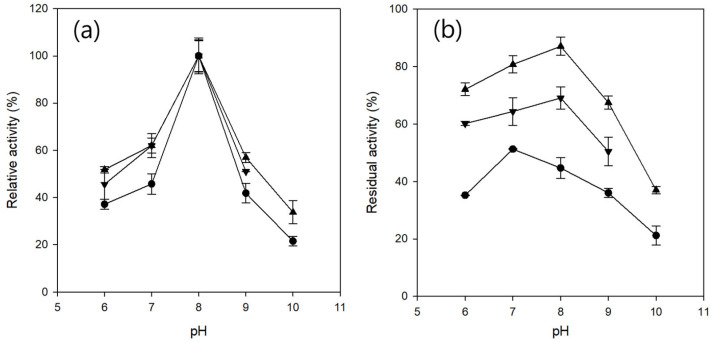
pH profile (**a**) and pH stability (**b**) at 25 °C of free CRL (●), CRL entrapped in Ba-alginate microbeads (▲), and CRL entrapped in Fe-alginate microbeads (▼). pH profiles were measured using sodium acetate buffer (pH 6), Tris-HCl buffer (pH 7–9), and Glycine-NaOH buffer (pH 10). pH stability was evaluated by measuring the residual activity after 12 h incubation at each pH at 25 °C. Fe-alginate microbeads were excluded at pH 10 due to instability.

The pH profiles of free CRL and CRL entrapped in Ba- and Fe-alginate microbeads were measured over a pH range of 6–10 (Figure 4a). All the samples exhibited maximum activity at pH 8. Activity declined more steeply in the alkaline range (pH 8–10) than in the acidic range (pH 6–8), indicating that CRL was more susceptible to deactivation under alkaline conditions. These results are consistent with those of previous reports on the pH sensitivity of CRL [46]. Across the tested range, the relative activity of the free CRL was consistently lower than that of its entrapped counterparts. CRL entrapped in Ba-alginate microbeads exhibited a slightly higher relative activity than CRL entrapped in Fe-alginate microbeads. In addition, the Fe-alginate microbeads could not be evaluated at pH 10 because the gel structure disintegrated and dissolved under strongly alkaline conditions.

The pH stability of free CRL and entrapped CRL was evaluated by measuring the residual activity after 12 h of incubation at each pH at 25 °C (Figure 4b). CRL entrapped in Ba-alginate exhibited the highest stability across the tested pH range, followed by CRL entrapped in Fe-alginate, whereas free CRL showed the lowest stability. Optimal pH stability for entrapped CRL was observed at pH 8, whereas free CRL was most stable at pH 7. Across all pH values, Fe-alginate microbeads exhibited lower residual activity than Ba-alginate microbeads, which may be attributed to higher enzyme leakage during incubation, possibly resulting from the higher swelling capacity of Fe-alginate gels, as described previously.

Notably, Fe-alginate microbeads were unstable at pH 10, which can be attributed to the reduced stability of Fe^3+^-alginate coordination under highly alkaline conditions. At elevated pH, the increased concentration of hydroxide ions can disrupt the coordination between Fe^3+^ and alginate carboxylate groups through ligand exchange or hydrolysis, rendering cross-linking sites more susceptible to displacement by competing cations (e.g., Na^+^) or chelating agents. Such displacement can promote Fe^3+^ release and lead to disintegration of the gel network [43]. In contrast, Ba-alginate forms a simpler two-dimensional “egg-box” structure that, despite its lower cross-linking density compared to Fe^3+^-alginate, appears more resistant to ionic displacement, thereby exhibiting superior pH stability.

In addition to the structural stability of the gel network, entrapment protects enzymes from pH-induced conformational changes [47]. The alginate matrix provides a confined microenvironment that buffers rapid protonation–deprotonation events, limits structural fluctuations, and reduces the exposure of the catalytic site to extreme pH conditions. Such conformational stabilization likely contributes to the higher pH tolerance of entrapped CRL compared to that of the free CRL, complementing the mechanical stability provided by the cross-linked gel.

Overall, these results reveal a trade-off between thermal stability and pH stability: Fe-alginate microbeads provide greater thermal resistance, whereas Ba-alginate microbeads offer superior stability under alkaline conditions, reflecting the intrinsic differences in cation-alginate binding mechanisms and network architecture.

#### 2.2.3. Kinetic Study of Entrapped CRL

Kinetic characterization is essential to understand how immobilization influences the catalytic behavior of enzymes. The Michaelis constant (K_m_), turnover number (k_cat_), and catalytic efficiency (k_cat_/K_m_) provide quantitative insights into the substrate affinity, catalytic rate, and overall performance, respectively. Immobilization can affect these parameters by introducing diffusion limitations, restricting enzyme conformational flexibility, and altering the physicochemical microenvironment of the active site. In this study, the hydrolysis of pNB by free and entrapped CRL was investigated, and kinetic constants were determined using the Lineweaver–Burk plot model.

The kinetic constants of free CRL and CRL entrapped in Ba- and Fe-alginate microbeads are summarized in Table 4. Among the samples, free CRL exhibited the lowest K_m_ (0.73 mM), followed by Ba-(0.88 mM) and Fe-alginate (2.30 mM). Because a lower K_m_ indicates higher substrate affinity, the increased values for the entrapped enzymes suggest reduced enzyme flexibility due to physical confinement within the microbead matrices. Ba-alginate microbeads displayed approximately three-fold higher substrate affinity than Fe-alginate microbeads, which may be attributed to differences in microbead size (341 µm vs. 383 µm) and swelling ratio (12.6 vs. 20.9). Larger microbeads with higher swelling capacity are more likely to impose diffusional restrictions, requiring substrate molecules to travel longer distances to reach the active sites, which may also enhance potential inhibitory effects [48]. Furthermore, the hydrophobic nature of pNB can hinder its diffusion into the hydrophilic alginate matrix, further increasing the apparent K_m_ by limiting substrate availability at the enzyme’s active site.

The turnover number (k_cat_)—which represents the number of substrate molecules converted per enzyme molecule per unit time—was highest for free CRL (1.01 × 10^4^ s^−1^), followed by Fe-alginate (2.53 × 10^3^ s^−1^) and Ba-alginate (1.07 × 10^3^ s^−1^). This reduction in k_cat_ upon entrapment is likely attributable to structural and microenvironmental changes rather than diffusional effects, because k_cat_ primarily reflects the chemical turnover step after substrate binding. Entrapment within the alginate network can restrict the conformational flexibility, particularly the lid-domain movement required for lipase activation, thereby slowing catalytic turnover [49]. Additionally, the hydrophilic nature of the alginate matrix may alter local polarity and hydration around the enzyme, potentially increasing the activation energy for the turnover of hydrophobic substrates such as pNB [50]. Notably, CRL entrapped in Fe-alginate exhibited a k_cat_ value approximately 2.4-fold higher than that of CRL entrapped in Ba-alginate, which may be related to morphological and structural differences. Consistent with the findings of Saxena et al. [51], the rice-shaped morphology of Fe-alginate microbeads may provide a more favorable enzymatic microenvironment, partially reducing the catalytic rate losses caused by entrapment.

Catalytic efficiency (k_cat_/K_m_), which integrates substrate binding affinity and catalytic turnover rate, provides a comprehensive measure of overall performance. Free CRL exhibited the highest catalytic efficiency, whereas CRL entrapped in Ba- and Fe-alginate retained approximately 8.7% and 7.9% of the catalytic efficiency of free CRL, respectively. This decrease can be attributed to the combined effects of increased apparent K_m_, from diffusional resistance and limited access of hydrophobic pNB to the active site within the hydrophilic gel matrix, and decreased k_cat_ from conformational restrictions and microenvironmental alterations. Although Ba-alginate entrapment preserved a higher substrate affinity, it suffered from a lower turnover rate, whereas Fe-alginate entrapment maintained a higher turnover rate but exhibited a substantially reduced substrate affinity. These opposing trends resulted in comparable overall catalytic efficiencies of the two entrapment systems.

## 3. Conclusions

This study demonstrated the successful fabrication of size-controlled alginate microbeads via an oil-free gas-shearing technique for CRL entrapment. By systematically optimizing the fabrication parameters, we established clear correlations between bead size (ranging from ~893 to ~247 µm), alginate concentration, and catalytic performance. Increasing the gas flow rate reduced the bead size and improved the mass transfer and activity retention, but sometimes at the expense of mechanical stability. Higher alginate concentrations enhanced bead integrity while slightly limiting the catalytic efficiency owing to diffusional resistance. Among the tested cross-linkers, Ba^2+^ and Fe^3+^ produced microbeads with superior immobilization yield, activity retention, and activity recovery compared with conventional Ca-alginate systems. CRL entrapped in Fe-alginate microbeads exhibited exceptional thermal stability, with a half-life at 60 °C approximately 127-fold greater than free CRL, 1.5-fold higher than CRL entrapped in Ba-alginate microbeads, as well as a 2.4-fold higher turnover number. In contrast, the CRL entrapped in Ba-alginate microbeads demonstrated greater pH stability across the tested range, higher substrate affinity, and better residual activity after repeated reuse, retaining more than 60% of its initial activity after five cycles. These contrasting behaviors are attributed to the distinct gel network architectures formed by divalent and trivalent cations, which modulate the enzyme microenvironment, mass transfer characteristics, and mechanical robustness.

Overall, the results confirmed that both Ba- and Fe-alginate microbeads are effective carriers for CRL entrapment, with complementary strengths that can be selected according to specific application requirements, such as thermal resistance or alkaline stability. The gas-shearing method provides a scalable, oil-free, and organic solvent-free platform for producing high-performance enzyme carriers with tunable properties, representing a practical and versatile approach for industrial biocatalysis. In addition, parameters such as bath temperature and gas pressure, although held constant in this study, could be further tuned to refine size control and enhance immobilization efficiency, offering additional opportunities to improve the versatility of this fabrication strategy.

## 4. Materials and Methods

### 4.1. Materials

Alginic acid sodium salt (from brown algae), glycine, CRL, and pNB were purchased from Sigma-Aldrich (St. Louis, MO, USA). Tris (base) was purchased from JT Baker (Phillipsburg, NJ, USA). Acetonitrile (ACN), isopropanol (IPA), calcium chloride, barium chloride, iron(III) chloride, acetic acid, sodium hydroxide, and methylene blue were purchased from Samchun Pure Chemicals (Pyeongtaek-si, Republic of Korea). Strontium chloride was purchased from Comscience (Gwangju-si, Republic of Korea). Magnesium chloride was purchased from Duksan Pure Chemicals (Ansan-si, Republic of Korea). Sodium acetate was purchased from Daejung Chemicals and Metals (Siheung-si, Republic of Korea).

### 4.2. Development of a Gas-Shearing Apparatus for Preparing Size-Controlled Alginate Hydrogel Microbeads

The gas-shearing apparatus was custom-designed based on the modifications of a previously reported study [22,23]. As shown in Figure 1, an inner needle (26 G; inner diameter: 0.260 mm) was inserted into the outer needle (18 G; inner diameter: 0.838 mm) to form a coaxial assembly. The outer needle is designed to align concentrically with the inner needle. Subsequently, an additional 18 G needle (side needle) was attached to the side of the outer needle to enable nitrogen gas injection. All needle junctions were sealed with resin. The nitrogen gas flow rate was controlled using a gas pressure regulator (General purpose single stage regulator, Victor-Torch Co., Plains, PA, USA) and a ball flow meter (Rate Master, Dwyer Instruments, Michigan City, IN, USA), whereas the volumetric flow rate of the solution was regulated using a split-type syringe pump (SPM, DK Infusetek, Shanghai, China). The apparatus setup is shown in Figure S2.

### 4.3. Entrapment of CRL in Alginate Microbeads via the Gas-Shearing Apparatus

Lyophilized CRL was dissolved in 3.5 mL of 50 mM Tris-HCl buffer (pH 7.2) in a 15 mL conical tube to a concentration of 30 mg/mL. The solution was incubated at 25 °C and 120 rpm for 10 min and centrifuged at 1200 rpm for 10 min. The resulting supernatant (protein content 5.9 mg/mL) was used as the lipase solution. Alginate/lipase mixtures with final alginate concentrations of 3%, 4%, and 5% (*w*/*v*) were prepared by mixing alginate stock solutions of 3.75%, 5.00%, and 6.25% (*w*/*v*), respectively, with the lipase solution at a 4:1 (*v*/*v*) ratio. Each mixture (10 mL; 8 mL alginate solution + 2 mL lipase solution) was incubated for 30 min. The final protein concentration was 1.2 mg/mL. Subsequently, an aliquot of 0.4 mL of the mixture was dropped into 50 mL of 1% (*w*/*v*) cross-linking solution (CaCl_2_, SrCl_2_, BaCl_2_, or FeCl_3_) at 40 μL/min for 10 min using a syringe pump, with a dispensing distance of 10 cm. The microbead size was controlled by adjusting the nitrogen gas flow rate between 0.5 and 2.0 L/min. The formed microbeads were cured by additional stirring in the cross-linking solution for 30 min, collected, and washed three times with Tris-HCl buffer (pH 7.2). The resulting microbeads were stored at 4 °C until use.

### 4.4. Physical Properties of CRL-Entrapped Alginate Microbeads

#### 4.4.1. Average Size

The average size and size distribution of the alginate microbeads were measured in triplicate using a particle size analyzer (Mastersizer 2000, Malvern Panalytical, Malvern, UK). The microbeads were dispersed in distilled water without sonication, using water as the dispersant (refractive index; RI = 1.330) and particle RI set to ~1.52.

#### 4.4.2. Microbead Morphology

To analyze the surface morphology of the alginate microbeads, the samples were frozen at −70 °C overnight and then freeze-dried under vacuum at −80 °C for 15 h. Before observation using a field-emission scanning electron microscope (SIGMA, Carl Zeiss, Oberkochen, UK), the freeze-dried microbeads were coated with platinum.

#### 4.4.3. Swelling Ratio

The swollen microbeads were gently blotted with Kimwipes to remove excess surface water and then weighed again. The microbeads were subsequently dried in a dry oven at 60 °C for 6 h, and their dry weights were measured. The swelling ratio (g/g dry weight) was calculated as follows:

(2) $$Swelling\;ratio=\frac{W_{t}-W_{d}}{W_{d}}$$

where $W_{t}$ and $W_{d}$ are the weights of freshly prepared alginate microbeads in the swollen state and after complete drying, respectively.

### 4.5. Characterization of CRL Entrapped in Alginate Microbeads

#### 4.5.1. Determination of Lipase Activity and Loaded Protein Content

The lipase activity was measured using a modified version of a previously reported method [26]. Lipase activity was measured using a spectrophotometric assay. For the CRL, 0.1 mL of the lipase solution was added to 9.4 mL of Tris-HCl buffer (pH 7.2). For entrapped CRL, one-fifth of the total number of microbeads prepared from 0.4 mL of the alginate/lipase mixture was dispersed in the same buffer (9.5 mL). The hydrolysis reaction was initiated by adding 0.5 mL of pNB (1 mM in IPA) and carried out at 25 °C and 120 rpm for 15 min. Samples were withdrawn every 5 min and mixed with ACN at a 1:1 ratio to stop the reaction. The mixtures were centrifuged, and the absorbance of the supernatant was measured at 400 nm to quantify the product p-nitrophenol using a standard calibration curve. The measured data were used to calculate the activity of CRL in units (U), where one unit is defined as the amount of enzyme that converts 1 μmol of substrate into product per minute under the specified conditions (U = μmol/min). All assays were performed in duplicate.

Protein quantification of CRL was performed using the Micro BCA™ Protein Assay Kit (Thermo Fisher Scientific, Waltham, MA, USA). To determine the amount of CRL entrapped within the alginate microbeads, the microbeads were recovered after curing, and the residual CRL in the cross-linking solution was quantified. The amount of entrapped CRL was calculated by subtracting the non-entrapped CRL in the cross-linking solution from the total CRL in the initial lipase solution. The immobilization yield, activity retention, and activity recovery were calculated using the following equations [26,27]:

(3) $$Immobilization\;yield\left(\%\right)=\frac{Immobilized\;enzyme\left(mg\right)}{Initial\;free\;enzyme\left(mg\right)}\times 100\left(\%\right)$$

(4) $$Activity\;retention\left(\%\right)=\frac{SA\;of\;immobilized\;enzyme\left(U/mg\right)}{SA\;of\;free\;enzyme\left(U/mg\right)}\times 100\left(\%\right)$$

(5) $$\mathrm{Activity}\;\mathrm{recovery}\left(\%\right)=\frac{Total\;activity\;of\;immobilized\;enzyme\left(U\right)}{Total\;activity\;of\;initial\;free\;enzyme(U)}\times 100\left(\%\right)$$

#### 4.5.2. Reusability of Entrapped CRL

To determine the reusability of the CRL entrapped in the alginate microbeads, the microbeads were used in an enzymatic reaction, followed by washing with Tris-HCl buffer (pH 7.2) three times by vacuum filtration. The washed microbeads were then reused for subsequent enzymatic reactions under identical conditions. To examine morphological changes after repeated use, optical microscopy was performed at 100× magnification using a light microscope (Olympus BX51, Olympus Co., Tokyo, Japan).

#### 4.5.3. Determination of Thermal Stability of Entrapped CRL

To evaluate the thermal stability of the CRL, 0.1 mL of free CRL solution (protein content of 5.9 mg/mL) was placed in a 50 mL conical tube containing 0.9 mL of Tris-HCl buffer (pH 7.2). For CRL entrapped in Ba- and Fe-alginate microbeads, one-fifth of the microbeads prepared from 0.4 mL of alginate/lipase solution were placed in a 50 mL conical tube containing 1 mL of the same buffer. The samples were incubated in a water bath at 60 °C and 80 rpm for 20 h. At designated time points, each tube was removed and immediately cooled in ice-cold water for 10 min. Residual activity was then measured at 25 °C. The half-life time (*t*_1/2_) was determined by fitting the data to a first-order deactivation kinetics using SigmaPlot 12.0.

(6) $$A_{t}=A_{0}e^{-kt}$$

(7) $$t_{1/2}=\frac{\ln2}{k}$$

where *t* is incubation time (min), *A_0_* is initial activity of CRL, *A_t_* is residual activity at time *t*, and *k* is the first-order deactivation rate constant of lipase (min^−1^). To check for enzyme leakage, supernatants collected after heating were assayed at 25 °C, and no detectable activity was observed, indicating that leakage did not affect residual activity measurements.

#### 4.5.4. Determination of pH Profile and pH Stability of Entrapped CRL

To determine the pH profile of lipase activity, 50 mM sodium acetate buffer (pH 6), 50 mM Tris-HCl buffer (pH 7–9), and 50 mM glycine-NaOH buffer (pH 10) were used. For free CRL, 0.1 mL of free CRL solution (protein content of 5.9 mg/mL) was added to 9.4 mL of each buffer, and the enzymatic reaction was carried out using the same method described above. For CRL entrapped in Ba- and Fe-alginate microbeads, the microbeads were washed three times with the respective pH buffers to equilibrate their internal pH. Subsequently, one-fifth of the microbeads prepared from 0.4 mL of alginate/lipase solution were dispersed in the corresponding buffer (9.5 mL), and the reaction was conducted under the same conditions. For each buffer condition, a blank reaction without lipase was performed using pNB, and its absorbance was used as background control.

To evaluate the pH stability, 0.1 mL of free CRL solution was added to 0.9 mL of each pH buffer, and CRL entrapped in Ba- and Fe-alginate microbeads was dispersed in each pH buffer (1 mL). The samples were incubated at 25 °C and 80 rpm for 12 h. After incubation, the residual activity was measured using the method described above.

#### 4.5.5. Kinetic Analysis of Entrapped CRL

To analyze the kinetic constants of pNB hydrolysis by free CRL and CRL entrapped in Ba- and Fe-alginate microbeads, the enzymatic reactions were conducted using pNB at concentrations ranging from 0.1 to 3.0 mM. The reaction was carried out in Tris-HCl buffer (pH 7.2) at 25 °C and 120 rpm for 15 min with sampling every 5 min. The data were fitted to the Lineweaver–Burk equation using SigmaPlot 12.0.

## Figures and Tables

**Table 1:** Effects of gas flow rate on the microbead size and various properties of entrapped CRL.

Gas Flow Rate (L/min) ^a^	Mean Diameter of Microbeads (μm)	Number of Microbeads	Immobilization Yield (%)	Activity Retention (%)	Activity Recovery (%)	Residual Activity After Last Reuse (%)	Maximum No. of Reuse Cycles
0.5	893 ^b^	122	44.9 ± 3.5	5.8 ± 0.2	2.6 ± 0.1	54.3 ± 3.9	2
0.8	576	681	36.2 ± 1.0	14.8 ± 1.1	5.3 ± 0.2	136.4 ± 3.0	1
1.0	482	2281	31.9 ± 2.4	17.7 ± 1.0	5.7 ± 0.7	ND ^c^	0
1.5	282	10,273	25.6 ± 3.7	21.5 ± 5.1	5.4 ± 0.5	ND	0
2.0	247	14,500	20.3 ± 2.6	29.6 ± 2.7	6.0 ± 1.3	ND	0

^a^ Experiments were conducted using 3% (*w*/*v*) alginate and cross-linked with 1% (*w*/*v*) CaCl_2_. ^b^ The mode value from the particle size analyzer was used because of the instrument’s inability to detect larger particles beyond its measurement range. ^c^ ‘Not Determined’ indicates microbead disintegration during the washing process, which rendered reuse impossible.

**Table 2:** Effects of alginate concentration on the activity recovery and reusability of entrapped CRL.

Alginate Concentration (%) ^a^	Mean Diameter of Microbeads (μm)	Activity Recovery (%)	Residual Activity After Last Reuse (%)	Maximum No. of Reuse Cycles
3.0	282	5.3 ± 0.5	ND ^b^	0
4.0	334	4.3 ± 0.6	ND	0
5.0	399	3.4 ± 0.1	299.5 ± 32.5	1

^a^ Microbeads were cross-linked with 1% (*w*/*v*) CaCl_2_ at a gas flow rate of 1.5 L/min. ^b^ ‘Not Determined’ indicates microbead disintegration during the washing process, which rendered reuse impossible.

**Table 3:** Effects of cross-linking metal ions on alginate microbead properties and CRL immobilization characteristics.

Crosslinking Metal Ion ^a^	Mean Diameter of Microbeads (μm)	Immobilization Yield (%)	Activity Retention (%)	Activity Recovery (%)	Residual Activity After 4th Reuse (%)	Maximum No. of Reuse Cycles	Swelling Ratio
Ca^2+^	399	44.8 ± 3.2	7.7 ± 0.3	3.4 ± 0.1	ND ^b^	1	16.4 ± 0.0
Sr^2+^	345	53.6 ± 4.2	8.9 ± 0.2	4.8 ± 0.3	77.7 ± 14.5	4	14.2 ± 0.0
Ba^2+^	341	57.8 ± 4.6	9.6 ± 0.7	5.5 ± 0.4	63.1 ± 11.8	>4	12.6 ± 0.0
Fe^3+^	383	67.1 ± 4.8	12.2 ± 0.7	8.2 ± 0.4	50.4 ± 0.8	>4	20.9 ± 0.0

^a^ Experiments were conducted using 5% (*w*/*v*) alginate cross-linked with 1% (*w*/*v*) metal ions at a gas flow rate of 1.5 L/min. ^b^ ‘Not Determined’ indicates microbead disintegration during the washing process, which rendered reuse impossible.

**Table 4:** Kinetic constants of free and entrapped CRL in the hydrolysis of pNB.

Kinetic Constant	Free CRL	CRL Entrapped in Ba-Alginate	CRL Entrapped in Fe-Alginate
K_m_ (mM)	0.726	0.884	2.296
k_cat_ (s^−1^)	1.01 × 10^4^	1.07 × 10^3^	2.53 × 10^3^
k_cat_/K_m_ (s^−1^ M^−1^)	1.39 × 10^7^	1.21 × 10^6^	1.10 × 10^6^

## Data Availability

The original contributions presented in this study are included in the article. Further inquiries can be directed to the corresponding author.

## Supplementary Materials

The following supporting information can be downloaded at https://www.mdpi.com/article/10.3390/gels11090710/s1. Figure S1: Particle size distribution of alginate microbeads prepared at different gas flow rates, measured using a particle size analyzer; Figure S2: (a) Setup of the gas-shearing apparatus mounted on a syringe pump and (b) gas-shearing needle.

## Abbreviations

The following abbreviations are used in this manuscript:

CRL	lipase from *Candida rugosa*
G-block	guluronic acid residues
ND	not detected
SA	specific activity
pNB	p-nitrophenyl butyrate
K_m_	Michaelis constant
k_cat_	turnover number
k_cat_/K_m_	catalytic efficiency
*t*_1/2_	half-life time
ACN	acetonitrile
IPA	isopropanol
