# Supplementary material for: Size-Controlled Fabrication of Alginate Hydrogel Microbeads Optimized for Lipase Entrapment
Source: Gels. 2025 Sep 4;11(9):710. doi: 10.3390/gels11090710 (PMC12469728; doi:10.3390/gels11090710)
Supplement: Supplementary file 1 [file gels-11-00710-s001.zip › gels-3854779-supplementary.pdf]

*Electronic supplementary information*

# Size-Controlled Fabrication of Alginate Hydrogel Microbeads Optimized for Lipase Entrapment

Dong Han Kim, Jeong Eun Cha, Dojin Kim and Sang Hyun Lee*

Advanced Materials Program, Department of Biological Engineering, Konkuk University, Seoul 05029, Republic of Korea

***Corresponding authors:**

Sang Hyun Lee

Tel.: +82-2-2049-6269

Fax: +82-2-457-8895

E-mail: sanghlee@konkuk.ac.kr

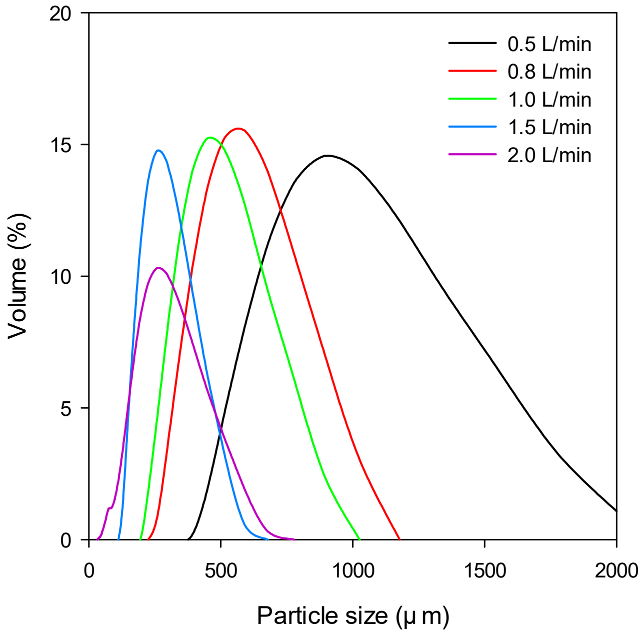


**Figure S1.** Particle size distribution of alginate microbeads prepared at different gas flow rates, measured using a particle size analyzer.

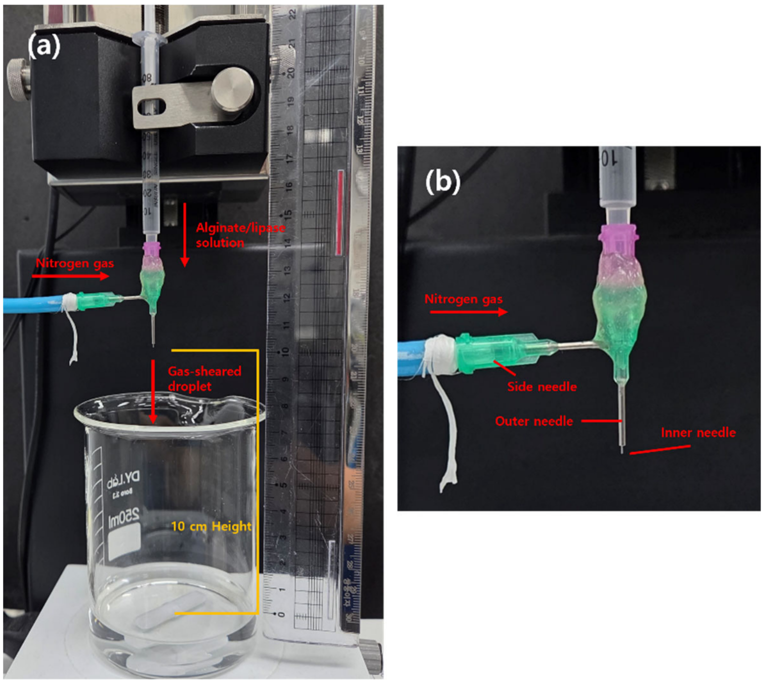


**Figure S2.** (a) Actual setup of the gas-shearing apparatus mounted on a syringe pump and (b) gas-shearing needle
